# PHYSICAL FRAILTY AS A PREDICTOR OF INCIDENT DISABILITY IN SPECIFIC-IADL ITEMS AMONG OLDER ADULTS

**DOI:** 10.1093/geroni/igac059.2483

**Published:** 2022-12-20

**Authors:** Jane Lee, Miji Kim, Chang Won Won

**Affiliations:** Yonsei University, Seoul, Seoul-t'ukpyolsi, Republic of Korea; Kyung Hee University, Seoul, Seoul-t'ukpyolsi, Republic of Korea; Kyung Hee University, Seoul, Seoul-t'ukpyolsi, Republic of Korea

## Abstract

This study investigated the 2-year impact of physical frailty on disability (activities of daily living [ADL], instrumental ADL [IADL], and mobility) and mortality among community-dwelling older adults in Korea. We used data from 2,905 older adults aged 70–84 years who participated in the Korean Frailty and Aging Cohort Study (KFACS) at baseline (2016–2017) and Wave 2 (2018–2019) with all five components of Fried’s physical frailty phenotype. Of these,277 (7.8 %) were frail and 1,312 (45.2 %) were robust. In the 2-year follow-up, multivariate analysis showed significant differences in frailty status for all disabilities (ADL, IADL, mobility) and mortality incidence. Both pre-frail (odds ratio [OR]=1.48, 95% confidence interval [CI]= 1.06–2.05) and frail (OR = 5.11, 95% CI=2.78–9.39) statuses showed increased risks of mobility disability. The incidence of ADL disability was significant only in frail older adults (OR =10.26, 95% CI=3.16–33.31). Both pre-frailty (OR =1.55, 95% CI=1.03–2.32) and frailty (OR =4.11, 95% CI=2.35–7.18) status were significantly associated with the incidence of IADL limitations. Frailty status was associated with disability in mobility-related items of IADL (going out, using transportation, and shopping) among men, and was associated with most IADL items among women after 2 years. The results of this study emphasize the need for sex-specific policies and frailty prevention programs, with a focus on detecting frailty before it leads to irreversible disability or other negative health outcomes. These findings may provide a framework for frailty prevention in community-dwelling older adults.

